# Regulation of Derlin-1-mediated degradation of NADPH oxidase partner p22^*phox*^ by thiol modification

**DOI:** 10.1016/j.redox.2022.102479

**Published:** 2022-09-13

**Authors:** Kei Miyano, Shuichiro Okamoto, Mizuho Kajikawa, Takuya Kiyohara, Chikage Kawai, Akira Yamauchi, Futoshi Kuribayashi

**Affiliations:** a Department of Natural Sciences, Kawasaki Medical School, 577 Matsushima Kurashiki, Okayama, 701-0192, Japan; b Department of Biochemistry, Kawasaki Medical School, 577 Matsushima Kurashiki, Okayama, 701-0192, Japan; c Laboratory of Microbiology, Showa Pharmaceutical University, 3-3165 Higashi-Tamagawagakuen, Machida, Tokyo, 194-8543, Japan; d Department of Medicine and Clinical Science, Graduate School of Medical Sciences, Kyushu University, 3-1-1 Maidashi, Higashi-ku, Fukuoka, 812-8582, Japan

**Keywords:** Chronic granulomatous disease, (CGD)/Derlin-1/, Endoplasmic reticulum-associated degradation, (ERAD)/NADPH oxidase/p22phox

## Abstract

The transmembrane protein p22^*phox*^ heterodimerizes with NADPH oxidase (Nox) 1–4 and is essential for the reactive oxygen species-producing capacity of oxidases. Missense mutations in the p22^*phox*^ gene prevent the formation of phagocytic Nox2-based oxidase, which contributes to host defense. This results in chronic granulomatous disease (CGD), a severe primary immunodeficiency syndrome. In this study, we characterized missense mutations in p22^*phox*^ (L51Q, L52P, E53V, and P55R) in the A22° type (wherein the p22^*phox*^ protein is undetectable) of CGD. We demonstrated that these substitutions enhanced the degradation of the p22^*phox*^ protein in the endoplasmic reticulum (ER) and the binding of p22^*phox*^ to Derlin-1, a key component of ER-associated degradation (ERAD). Therefore, the L^51^-L^52^-E^53^-P^55^ sequence is responsible for protein stability in the ER. We observed that the oxidation of the thiol group of Cys-50, which is adjacent to the L^51^-L^52^-E^53^-P^55^ sequence, suppressed p22^*phox*^ degradation. However, the suppression effect was markedly attenuated by the serine substitution of Cys-50. Blocking the free thiol of Cys-50 by alkylation or C50S substitution promoted the association of p22^*phox*^ with Derlin-1. Derlin-1 depletion partially suppressed the degradation of p22^*phox*^ mutant proteins. Furthermore, heterodimerization with p22^*phox*^ (C50S) induced rapid degradation of not only Nox2 but also nonphagocytic Nox4 protein, which is responsible for redox signaling. Thus, the redox-sensitive Cys-50 appears to determine whether p22^*phox*^ becomes a target for degradation by the ERAD system through its interaction with Derlin-1.

## Highlights

• Missense mutations in exon 3 of p22^*phox*^ enhance the binding of p22^*phox*^ to Derlin-1.
• Oxidation of the thiol group of p22^*phox*^ Cys50 suppresses p22^*phox*^ degradation.
• Serine substitution of Cys-50 increases the affinity of p22^*phox*^ for Derlin-1.
• Stability of the p22^*phox*^ protein is regulated by redox-sensitive Cys-50.

## Graphical abstract

**Figure:**
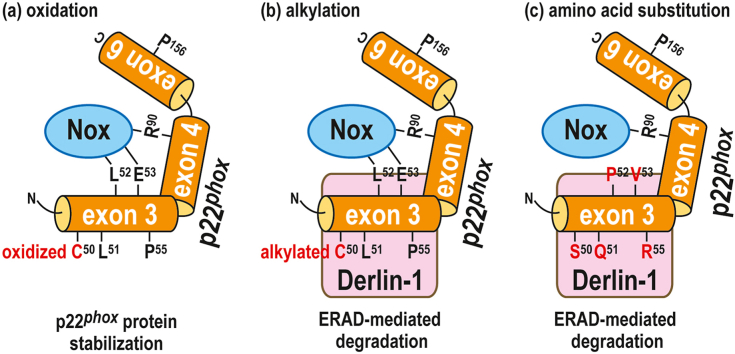

## 1 Introduction

The NADPH oxidase (Nox) family of enzymes produces reactive oxygen species (ROS) [[1], [2], [3], [4], [5]]. This family participates in variety biological functions, including host defense [1], signal transduction [6], otoconia synthesis [7], and hormone synthesis [8]. The human Nox family comprises seven members (Nox1–5, Duox1, and Duox2). Among the Nox family members, Nox2 (a.k.a. gp91^*phox*^) is the prototype and is expressed abundantly in professional phagocytes (e.g., neutrophils and macrophages), where it contributes to host defense by generating substantial quantities of superoxide. The superoxide generated is the precursor for other ROS (highly reactive), including hydrogen peroxide and hydroxyl radicals, which are involved in bacterial killing. Genetic defects in Nox2 (encoded by the X-linked *CYBB* gene) lead to chronic granulomatous disease (CGD), which is characterized by recurrent life-threatening bacterial and fungal infections [9].

The Nox partner protein p22^*phox*^ is essential for Nox1–Nox4-based oxidase activity [2]. The multiple membrane-spanning protein p22^*phox*^ heterodimerizes with the multiple membrane-spanning proteins Nox1–4, except Nox5. *De novo* p22^*phox*^ interacts with *de novo* Nox2 in the endoplasmic reticulum (ER) [[10], [11], [12]]. Nox2 immediately exits the ER and reaches the phagocyte/plasma membrane in a heterodimerization-dependent manner [10]. In the absence of p22^*phox*^, the Nox2 monomer is degraded by ER-associated degradation (ERAD) [10,13,14]. This explains why Nox2 protein is undetectable in the background of p22^*phox*^ (*CYBA* gene) genetic deficiency [[15], [16], [17]]. Thus, heterodimerization with p22^*phox*^ appears to promote proper the folding of Nox2 to evade degradation by ERAD.

Nonphagocytic Nox4 oxidase is expressed abundantly in endothelial cells (ECs) of blood vessels [18] and contributes to redox signaling, leading to changes in physiological processes, such as angiogenesis [19]. Unlike Nox2, Nox4 is primarily localized in the ER, where it also interacts with p22^*phox*^ [[20], [21], [22]]. The presence of p22^*phox*^ is also required for the detection of Nox4 protein. We previously reported that in transformed ECs, Nox4 protein levels are attenuated by the hypoxia-induced reduction of p22^*phox*^ mRNA and protein levels [23]. In addition, Nox4 was undetectable in an animal model expressing the p22^*phox*^ (Y121H) mutant protein with reduced protein expression instead of wild-type p22^*phox*^ [22,24].

The heterodimerization with p22^*phox*^ is indispensable for the localization of Nox2 in the phagocyte/plasma membrane. In addition, p22^*phox*^ functions as an anchor for the soluble cytosolic activating protein p47^*phox*^, forming an active complex. The formation of a complex with Nox2 occurs through the interaction between p47^*phox*^ and p22^*phox*^, because p47^*phox*^ forms a ternary complex with other activating proteins—p67^*phox*^ and p40^*phox*^. A missense mutation in *CYBA* (p22^*phox*^ gene), which results in an amino acid substitution of glutamine for proline-156, impairs the binding of p22^*phox*^ to p47^*phox*^ [25,26]. Because the expression of p22^*phox*^ is responsible for Nox2 localization and activation, genetic defects and missense mutations in p22^*phox*^ also cause CGD.

The interaction with p22^*phox*^ is also required for Nox4 activity [20]. The activity of Nox4 is independent of the presence of Nox-activating proteins, such as p47^*phox*^ and p67^*phox*^. The amount of ROS generated by Nox4 is proportional to the expression levels of Nox4–p22^*phox*^. A switch for activating Nox2 is turned off by the dissociation of p22^*phox*^ and the soluble cytosolic activating protein p47^*phox*^, whereas a switch for Nox4 activity is not readily turned off. To the best of our knowledge, a switch mechanism for the enzymatic activity of Nox4 has not been proposed yet.

A missense mutation in p22^*phox*^ (P156Q) has been functionally characterized [25,26]. The mutated proteins are unable to bind to p47^*phox*^ [25,26]. Thus, Pro-156 is considered to be responsible for the activation of Nox2. The mutational hotspot located in *CYBA* exon 3 in the A22° type (wherein the p22^*phox*^ protein is undetectable) of CGD exhibits missense mutations in p22^*phox*^ (L51Q, L52P, E53V, and P55R) [16]; however, to the best of our knowledge, these mutations have not been characterized yet. In the present study, we characterized missense mutations in p22^*phox*^ (L51Q, L52P, E53V, and P55R) and demonstrated that these amino acid substitutions promote the degradation of p22^*phox*^ protein in the ER. Interestingly, all of the mutant proteins strongly promoted the binding of p22^*phox*^ to Derlin-1, a key component of the ERAD system [[27], [28], [29], [30]]. These findings suggest that these amino acids (Leu-51, Leu-52, Glu-53, and Pro-55) are responsible for the stability of p22^*phox*^ protein in the ER. Furthermore, the L52P and E53V substitutions impaired the binding of p22^*phox*^ to Nox2. Thus, the L^51^-L^52^-E^53^-P^55^ sequence is involved in Nox2-based oxidase activity through a mechanism different from that of Pro-156.

We further demonstrated that the stability of the p22^*phox*^ protein is regulated by redox-sensitive Cys-50, which is adjacent to the L^51^-L^52^-E^53^-P^55^ sequence, in a thiol oxidation-dependent manner. A C50S substitution results in decreased protein stability. Moreover, blocking the free thiol of Cys-50 by alkylation or C50S substitution promoted the association of p22^*phox*^ and Derlin-1. The Nox2 and Nox4 proteins form a complex with p22^*phox*^ (C50S) and are rapidly degraded. Thus, the C-terminal region adjacent to Cys-50 (amino acids 50–55, including Cys-50) appears to be responsible for the stability of p22^*phox*^ and its partners, Nox2 and Nox4. Because the activity and stability of Nox4 are dependent on the presence of p22^*phox*^ [23], we propose that p22^*phox*^ degradation resulting from the modification of Cys-50 thiol group is a switch that turns off Nox4 activity.

## 2 Materials and methods

### 2.1 Materials

*Chemicals, reagents, and antibodies:* All general ultrapure-grade reagents were purchased from Nacalai Tesque (Kyoto, Japan), Wako Pure Chemicals Industries (Tokyo, Japan), or Sigma-Aldrich Japan (Tokyo, Japan), unless otherwise stated. Primers were purchased from Eurofins Genomics (Tokyo, Japan). Methyl-PEG_24_-maleimide reagent (polyethylene glycol [PEG]-maleimide) (catalog #22713) was purchased from ThermoFisher Scientific (Tokyo, Japan). The following antibodies were used: mouse monoclonal antibodies against β-tubulin (Wako, 10G10), Myc (Santa Cruz Biotechnology, 9E10), FLAG (DYKDDDDK-tag) (Wako, 1E6), and p22^*phox*^ (Santa Cruz Biotechnology, CS9); rabbit monoclonal antibody against protein disulfide isomerase (PDI; Cell Signaling Technology, C81H6); and rabbit polyclonal antibodies against p22^*phox*^ (GeneTex, GTX133970), Nox4 (GeneTex, N3C3), β-actin (Cell Signaling Technology, catalog #4967), and Derlin-1 (Medical & Biological Laboratories, PM018). All antibodies were used at a 1000-fold dilution.

*Plasmids and cDNA:* Sequences encoding peptide epitopes (“FLAG–(Gly)_3_–FLAG–(Gly)_3_” or “Myc–(Gly)_3_–Myc–(Gly)_3_”) were inserted into pcDNA3.1 for expression in mammalian cells. A modified vector, termed pcDNA3.1–FLAG or pcDNA3.1–Myc, was used to insert a FLAG tag at the N- or C-terminus of the protein or a Myc tag at the N- or C-terminus of the protein, respectively [21,31]. cDNAs encoding human p22^*phox*^, Nox2, Nox4, p47^*phox*^, and p67^*phox*^ were prepared as described previously and ligated to pcDNA3.1 [21,[31], [32], [33]]. The cDNAs for human Derlin-1 were prepared through RT-PCR using mRNA from EA.hy926 cells. Mutations leading to the indicated substitution were introduced by polymerase chain reaction-mediated, site-directed mutagenesis. All the constructs were sequenced for confirmation of their identities.

### 2.2 Cells, cell culture, and plasmid transfection

The CHO–K1 or HeLa cells were cultured as described previously [31,33]. The plasmids were transfected into CHO–K1 or HeLa cells as previously described [31,33].

### 2.3 Estimation of protein expression levels: sample preparation for immunoblotting and immunoblotting procedure

The samples were prepared as described previously [33]. Immunoblotting was performed as described previously [33]. Rabbit polyclonal antibodies against p22^*phox*^ (GTX133970), Nox4 (GeneTex, N3C3) and Derlin-1 (MBL, PM018) were used to detect p22^*phox*^–Myc, FLAG–Nox4 and Derlin-1, respectively; a mouse monoclonal antibody against FLAG (1E6) was used to detect FLAG–Nox2 and FLAG–Nox4; and mouse monoclonal antibodies against Myc (9E10) and β-tubulin (10G10) were used to detect 22^*phox*^–Myc and β-tubulin, respectively.

### 2.4 Determination of the oxidation state of p22^phox^

The transfected CHO–K1 cells (7 × 10^5^ cells in 6-well plates) were treated with or without 20 mM N-ethylmaleimide (NEM) or 1 mM H_2_O_2_ for 30 min. The treated and untreated cells were washed with phosphate-buffered saline (PBS) (137 mM NaCl, 2.68 mM KCl, 8.1 mM Na_2_HPO_4_, and 1.47 mM KH_2_PO_4_, pH 7.4) and lysed using 50 μl of 50 mM PEG-maleimide in lysis buffer (20 mM Tris–HCl, pH 7.4, 150 mM NaCl, 1% Triton X-100, and 1% (v/v) protease inhibitor cocktail). The lysates were incubated for 1 h at 4 °C and centrifuged for 20 min at 17,000×*g*. The supernatants were mixed with reducing sodium dodecyl sulfate (SDS)-sample buffer [1% (v/v) 2-mercaptoethanol]. The samples were then analyzed through SDS–polyacrylamide gel electrophoresis and immunoblotted with either anti-p22^*phox*^ antibody or anti-Myc antibody.

### 2.5 Immunofluorescence microscopy

Immunofluorescence microscopy was performed as described previously [21]. Briefly, to stain p22^*phox*^–Myc, Derlin-1–FLAG and PDI (ER marker), plasmid-transfected CHO–K1 cells grown on coverslips were fixed for 15 min in 4% formaldehyde at room temperature and then for 10 min in ice-cold 100% methanol at −20 °C, followed by permeabilization for 60 min in 0.3% Triton X-100 in PBS with 5% bovine serum albumin (BSA). The samples were incubated overnight at 4 °C with the indicated primary antibodies in PBS with 1% BSA and 0.3% Triton X-100; subsequently, these samples were incubated for 1–2 h at room temperature with secondary antibodies in PBS with 1% BSA and 0.3% Triton X-100. Mouse monoclonal antibodies against p22^*phox*^ (CS9) or Myc tag (9E10) were used to detect p22^*phox*^ (green), mouse monoclonal antibodies against FLAG (1E6) was used to detect Derlin-1–FLAG (magenta), a rabbit monoclonal antibody against PDI (C81H6) was used to detect the ER marker PDI (magenta) and Hoechst staining was used to detect cell nuclei (blue).

### 2.6 Assay of O_2_^−^ or H_2_O_2_ production

The production of O_2_^−^ by cells expressing Nox2 was assayed using Diogenes-luminol solution as described previously [31]. Briefly, the transfected cells (7 × 10^5^ cells in 6-well plates) were cultured for 24 h and harvested by incubation with trypsin/ethylenediaminetetra-acetic acid. After being washed with PBS, the cells were suspended at a density of 7 × 10^5^ cells per 250 μl PBS plus 10 μl Diogenes-luminol solution. The cells were treated with 200 ng/ml phorbol 12-myristate 13-acetate and then transferred to 96-well plates with white walls and flat bottoms (IWAKI, 3620–096). Using a spectral scanning multimode reader (Varioskan® Flash, Thermo), chemiluminescence was measured for 25 min at 37 °C with or without 2 μg/ml superoxide dismutase.

The production of H_2_O_2_ by cells expressing Nox4 was assayed using the homovanillic acid–horseradish peroxidase detection system as described previously [21,31].

### 2.7 Protein stabilization

Protein stability in plasmid-transfected CHO–K1 cells and HeLa cells was analyzed as described previously [21]. Briefly, the transfected cells were treated with 10 μg/ml cycloheximide (CHX) for the indicated times. When p22^*phox*^ was coexpressed with Nox2 or Nox4, the transfected cells were exposed to 10 μg/ml Brefeldin A for 1 h and then treated with 10 μg/ml cycloheximide (CHX) in the presence or absence of 20 μM MG132. The Brefeldin A treatment was performed to inhibit ER exit of the Nox–p22^*phox*^ complex to estimate the stability of the complex in the ER. To estimate the protein levels, the band intensities observed in the immunoblotting experiment were assessed using ImageJ software (National Institutes of Health, Bethesda, MD, USA).

### 2.8 Immunoprecipitation assay

Immunoprecipitation assay was performed as described previously [ [21]]. Briefly, plasmid-transfected CHO–K1 cells or HeLa cells (2.1 × 10^6^ in a 6-cm dish) were lysed using lysis buffer (20 mM Tris–HCl, pH 7.4; 150 mM NaCl; 1% Triton X-100). For transfected CHO cells, proteins in the lysates were precipitated with ANTI-FLAG® M2 Agarose Affinity Gel or Mouse IgG–Agarose (Sigma-Aldrich). For the transfected HeLa cells, proteins in the lysates were precipitated by anti-Myc antibody or control IgG (Fig. 10A).

### 2.9 Cell surface biotinylation assay

Cell surface biotinylation assay was performed as described previously [31].

### 2.10 Derlin-1 knockdown

The knockdown of Derlin-1was performed as described previously [21,31]. HeLa cells were cultured as described previously [21]. Briefly, the following 25-nucleotide modified synthetic double-stranded siRNA targeting Derlin-1 (Stealth RNAi) was purchased from Invitrogen: Derlin-1 siRNA, 5′-GAGAGGAGGAGUAUCAGGAUUUGGU-3′ (sense) and 5′-ACCAAAUCCUGAUACUCCUCCUCUC-3′ (antisense). Stealth RNAi negative-control duplexes (Invitrogen) were used as the negative control. HeLa cells were cultured in 6-well plates (1 × 10^5^ in a well) and then transfected with 200 pmol RNA using Lipofectamine RNAiMAX (ThermoFisher Scientific) in 50 μl of Opti-MEM (ThermoFisher Scientific) according to the manufacturer's instructions. Stealth RNA-transfected cells were cultured for 48 h and then transfected with the following plasmids: pcDNA3.1-wild-type (wt) p22^*phox*^–Myc (0.1 μg), pcDNA3.1-p22^*phox*^ (L51Q)–Myc (0.1 μg), pcDNA3.1-p22^*phox*^ (L52P)–Myc (0.1 μg), pcDNA3.1-p22^*phox*^ (E53V)–Myc (0.1 μg), pcDNA3.1-p22^*phox*^ (P55R)–Myc (0.1 μg), or pcDNA3.1-p22^*phox*^ (C50S)–Myc (0.1 μg). The transfected cells were cultured for 24 h and used to measure p22^*phox*^ protein levels.

### 2.11 Statistical analysis

Data were expressed as mean ± standard deviations. Between-group comparisons were performed using *t*-test and Tukey–Kramer multiple comparison of means test. Statistical analyses were performed using EZR (Saitama Medical Center, Jichi Medical University, Saitama, Japan), a graphical user interface for R (The R Foundation for Statistical Computing, Vienna, Austria).

## 3 Results

### 3.1 Characterization of p22^phox^ CGD mutants harboring mutations in the amino acid sequence that corresponds to exon 3

**Fig. 1:**
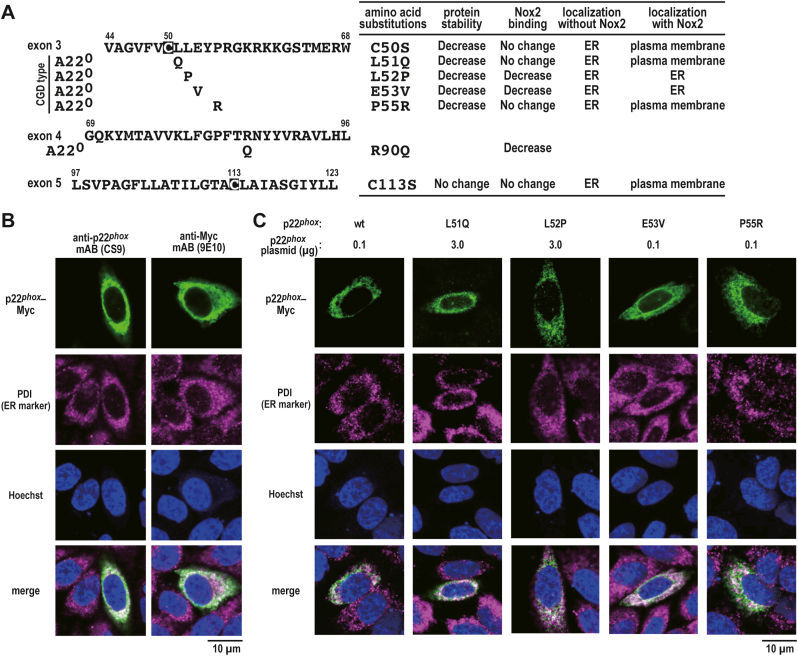
**p22**^***phox***^**CGD mutant proteins in this study** *A*, amino acid sequence that corresponds to exons 3 and 5 in p22^*phox*^. A22° is the CGD phenotype. The “A” letter and “22” number refer to autosomal recessive and p22^*phox*^ and the superscript ° indicates whether the p22^*phox*^ protein is absent based on immunoblotting [16]. Five missense mutations are located in exon 3 (L51Q, L52P, E53V, and P55R) and exon 4 (R90Q). *B and C*, distribution of exogenous wild-type p22^*phox*^ or mutant p22^*phox*^ in CHO–K1 cells. CHO–K1 cells (7 × 10^5^ cells in a 30-mm glass-bottom dish) were transfected with the indicated plasmids: pcDNA3.1-wild-type (wt) p22^*phox*^–Myc (0.1 μg), pcDNA3.1-p22^*phox*^ (L51Q)–Myc (3.0 μg), pcDNA3.1-p22^*phox*^ (L52P)–Myc (3.0 μg), pcDNA3.1-p22^*phox*^ (E53V)–Myc (0.1 μg), or pcDNA3.1-p22^*phox*^ (P55R)–Myc (0.1 μg). After fixation, the immunofluorescence signals were observed by confocal microscopy. Scale bars, 10 μm. These experiments have been repeated more than three times with similar results.

We evaluated four amino acid residues (Leu-51, Leu-52, Glu-53, and Pro-55) in the amino acid sequence that corresponds to exon 3 (Fig. 1A) based on the fact that the L51Q, L52P, E53V, and P55R mutations lead to the A22° type of CGD [16]. Because the amino acid substitutions may interfere with the recognition of p22^*phox*^ mutant proteins by anti-p22^*phox*^ mouse monoclonal antibody [mAB (CS9)] or rabbit polyclonal antibody, wild-type (wt) p22^*phox*^ and mutant proteins were prepared with a C-terminal Myc tag (p22^*phox*^–Myc), which is detectable with mAB 9E10 (anti-Myc mouse monoclonal antibody).

**Fig. 2:**
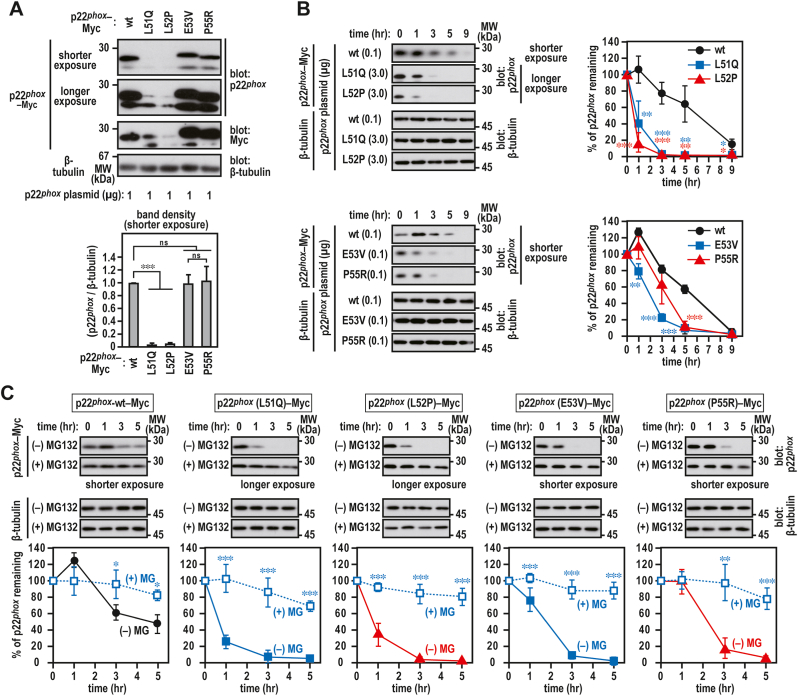
**Stability of p22**^***phox***^**CGD mutant proteins** *A*, expression levels of p22^*phox*^ mutant protein. CHO–K1 (7 × 10^5^ cells in 6-well plates) cells were transfected with the indicated plasmids: pcDNA3.1-wild-type (wt) p22^*phox*^–Myc (1 μg), pcDNA3.1-p22^*phox*^ (L51Q)–Myc (1 μg), pcDNA3.1-p22^*phox*^ (L52P)–Myc (1 μg), pcDNA3.1-p22^*phox*^ (E53V)–Myc (1 μg), or pcDNA3.1-p22^*phox*^ (P55R)–Myc (1 μg). *B and C,* stability of p22^*phox*^ mutant protein. CHO–K1 cells (7 × 10^5^ cells in 6-well plates) were transfected with the indicated plasmids: pcDNA3.1-wild-type (wt) p22^*phox*^–Myc (0.1 μg), pcDNA3.1-p22^*phox*^ (L51Q)–Myc (3.0 μg), pcDNA3.1-p22^*phox*^ (L52P)–Myc (3.0 μg), pcDNA3.1-p22^*phox*^ (E53V)–Myc (0.1 μg), or pcDNA3.1-p22^*phox*^ (P55R)–Myc (0.1 μg). The transfected cells were treated for 0, 1, 3, 5, or 9 h with cycloheximide (CHX) in the presence or absence of 20 μM MG132. Protein levels of the indicated proteins were estimated via immunoblotting. Positions for marker proteins are indicated in kDa. Each graph represents the relative density of the bands normalized to β-tubulin (n = 3). Statistical analysis was performed using Tukey–Kramer test. ***, *p* < 0.001; **, *p* < 0.05; *, *p* < 0.05; ns, no significance; shorter exposure, shorter exposure films were used for scanning; longer exposure, longer exposure films were used for scanning. These experiments have been repeated more than three times with similar results.

When wild-type p22^*phox*^–Myc was ectopically expressed alone in CHO–K1 cells, which do not express endogenous p22^*phox*^ and Nox2 [34], wild-type p22^*phox*^–Myc was observed to be colocalized with the ER marker protein PDI under a confocal laser microscope (Fig. 1B). The mutant proteins (L51Q, L52P, E53V, and P55R) were also colocalized with PDI (Fig. 1C). The laser intensity was adjusted to capture the images for the p22^*phox*^ (L51Q) and p22^*phox*^ (L52P) mutant proteins with low expression as described in detail below (Fig. 2). Thus CHO–K1 cells expressing exogenous p22^*phox*^ proteins represent a useful system to characterize p22^*phox*^ mutant proteins in the ER.

When CHO–K1 cell lysates expressing exogenous p22^*phox*^–Myc proteins were immunoblotted with polyclonal antibody to p22^*phox*^ and monoclonal antibody (9E10) to the Myc tag, the L51Q and L52P substitutions resulted in a decrease in the amount of the mutant protein (Fig. 2A). This result suggests that the mutation makes the protein unstable.

We investigated the effects of amino acid substitutions on the stability of p22^*phox*^. CHO K1 cells expressing p22^*phox*^–Myc were treated with cycloheximide (CHX) to inhibit the *de novo* synthesis of p22^*phox*^ in time course experiments (0, 1, 3, 5, and 9 h). The resulting cell lysates were analyzed by immunoblotting. The levels of p22^*phox*^ (L51Q)–Myc and p22^*phox*^ (L52P)–Myc mutant proteins were decreased to approximately 40% and 20%, respectively, after exposure to CHX for 1 h (Fig. 2B). In contrast, the E53V and P55R substitutions exerted no effect on protein expression (Fig. 2A). The levels of p22^*phox*^ (E53V)–Myc and p22^*phox*^ (P55R)–Myc mutant proteins were maintained approximately 80% and 100%, respectively, after exposure to CHX for 1 h. However, these substitutions also affected the levels of p22^*phox*^ mutant proteins after exposure to CHX for 5 h (Fig. 2B). These results suggest that these amino acid residues are responsible for the stability of p22^*phox*^ protein in the ER. The degradation of the mutant proteins was considerably suppressed in the presence of MG132 (Fig. 2C), indicating that proteasome is responsible for p22^*phox*^ degradation.

**Fig. 3:**
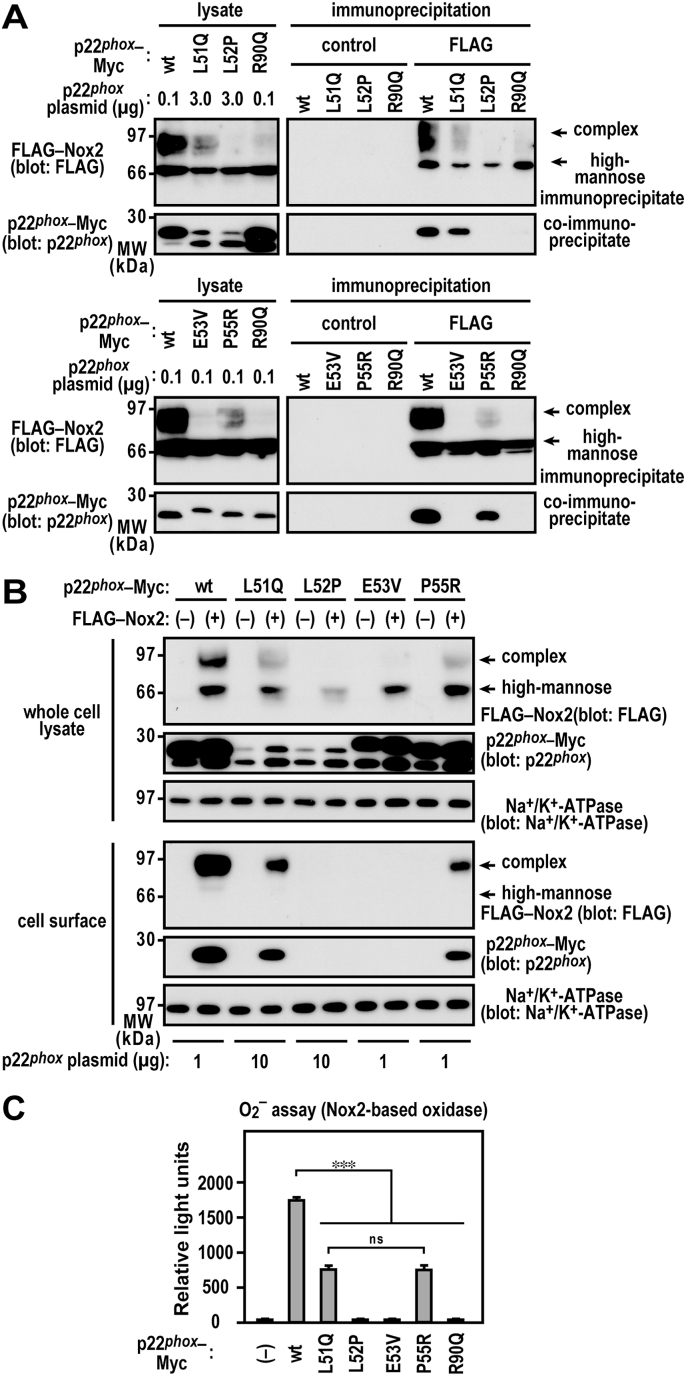
**Analysis of p22**^***phox***^**CGD mutant proteins** *A*, interaction of p22^*phox*^ with Nox2. CHO–K1 cells (2.1 × 10^6^ in a 6-cm dish) were transfected simultaneously with the indicated plasmids: pcDNA3.1-wild-type (wt) p22^*phox*^–Myc (0.1 μg), pcDNA3.1-p22^*phox*^ (L51Q)–Myc (3.0 μg), pcDNA3.1-p22^*phox*^ (L52P)–Myc (3.0 μg), pcDNA3.1-p22^*phox*^ (E53V)–Myc (0.1 μg), pcDNA3.1-p22^*phox*^ (P55R)–Myc (0.1 μg), pcDNA3.1-p22^*phox*^ (R90Q)–Myc (0.1 μg), and/or pcDNA3.1-FLAG–Nox2 (3.0 μg). control, Mouse IgG–Agarose; FLAG, ANTI-FLAG® M2 Agarose Affinity Gel. *B*, cell surface localization of p22^*phox*^ with Nox2. CHO–K1 cells (3.2 × 10^6^ in a 9-cm dish) were transfected simultaneously with the indicated plasmids: pcDNA3.1-wild-type (wt) p22^*phox*^–Myc (1 μg), pcDNA3.1-p22^*phox*^ (L51Q)–Myc (10 μg), pcDNA3.1-p22^*phox*^ (L52P)–Myc (10 μg), pcDNA3.1-p22^*phox*^ (E53V)–Myc (1 μg), pcDNA3.1-p22^*phox*^ (P55R)–Myc (1 μg), and/or pcDNA3.1-FLAG–Nox2 (9 μg). *C*, superoxide production by Nox2. CHO–K1 cells (7 × 10^5^ cells in 6-well plates) were transfected simultaneously with the indicated plasmids: pcDNA3.1-wild-type (wt) p22^*phox*^–Myc (0.03 μg), pcDNA3.1-p22^*phox*^ (L51Q)–Myc (0.3 μg), pcDNA3.1-p22^*phox*^ (L52P)–Myc (0.3 μg), pcDNA3.1-p22^*phox*^ (E53V)–Myc (0.03 μg), pcDNA3.1-p22^*phox*^ (P55R)–Myc (0.03 μg), pcDNA3.1-p22^*phox*^ (R90Q)–Myc (0.03 μg), pcDNA3.1-FLAG–Nox2 (1 μg), pcDNA3.1-Myc–p67^*phox*^ (0.2 μg), and/or pcDNA3.1-Myc–p47^*phox*^ (0.2 μg). Superoxide production was assayed using superoxide dismutase inhibitable-chemiluminescence using Diogenes. Each graph represents the mean ± standard deviation of the chemiluminescence intensities integrated for 10 min after PMA stimulation from three independent transfections. ***p < 0.001; ns, no significance. Protein levels of the indicated proteins were estimated via immunoblotting. Positions for marker proteins are indicated in kDa. Each graph represents the relative density of the bands normalized to β-tubulin (n = 3). complex, complex *N*-glycan-bearing Nox2; high-mannose, high mannose *N*-glycan-bearing Nox2. These experiments have been repeated more than three times with similar results.

In addition to L51Q, L52P, E53V, and P55R, a missense mutation in p22^*phox*^ (R90Q) (A22° type CGD) has not been characterized functionally in Nox2-based oxidase. A previous study suggested that this substitution impaired the interaction of p22^*phox*^ with Nox4 [35]. To determine whether p22^*phox*^ mutant proteins bind to Nox2, we expressed wild-type p22^*phox*^–Myc or p22^*phox*^–Myc mutant proteins together with FLAG–Nox2 (the FLAG tag was inserted at the N-terminus of Nox2). When the FLAG–Nox2 proteins were immunoprecipitated from the cell lysates of the transfected CHO–K1 cells, Nox2 did not coprecipitate p22^*phox*^ (R90Q)–Myc (Fig. 3A). The L52P and E53V mutations considerably impaired the binding of p22^*phox*^ to Nox2 to an extent same as that of the R90Q mutation. Using a cell surface biotinylation assay, we demonstrated that p22^*phox*^ (L51Q) and p22^*phox*^ (P55R), but not p22^*phox*^ (L52P) and p22^*phox*^ (E53V), localize at the plasma membrane in a Nox2 coexpression-dependent manner (Fig. 3B). In addition, complex *N*-glycan–bearing Nox2 (cell surface-localized form) was detected on the plasma membrane when wild-type p22^*phox*^, p22^*phox*^ (L51Q), or p22^*phox*^ (P55R) was coexpressed. Conversely, the high-mannose form of Nox2 (ER-localized form) was only detected in the lysate coexpressing p22^*phox*^ (L52P) or p22^*phox*^ (E53V), which cannot bind to Nox2 (Fig. 3A and B). To examine whether Nox2–p22^*phox*^ complexes generate superoxide extracellularly, we expressed wild-type p22^*phox*^–Myc or p22^*phox*^–Myc mutant proteins together with a set of FLAG–Nox2, p67^*phox*^, and p47^*phox*^. Under the same expression conditions that was used for wild-type p22^*phox*^, p22^*phox*^ (R90Q) failed to support superoxide production by Nox2, whereas the production was partially supported by the expression of p22^*phox*^ (L51Q)–Myc and p22^*phox*^ (P55R)–Myc (Fig. 3C). However, as the complex *N*-glycan-bearing Nox2 was not detected in the cell surface fraction coexpressing p22^*phox*^ (L52P) or p22^*phox*^ (E53V), which cannot bind to Nox2 (Fig. 3A), superoxide generation was not observed (Fig. 3C). These results suggest that a region corresponding to exon 3 is involved in the heterodimerization of p22^*phox*^ with Nox2.

### 3.2 Redox-sensitive cysteine residues in p22^phox^

**Fig. 4:**
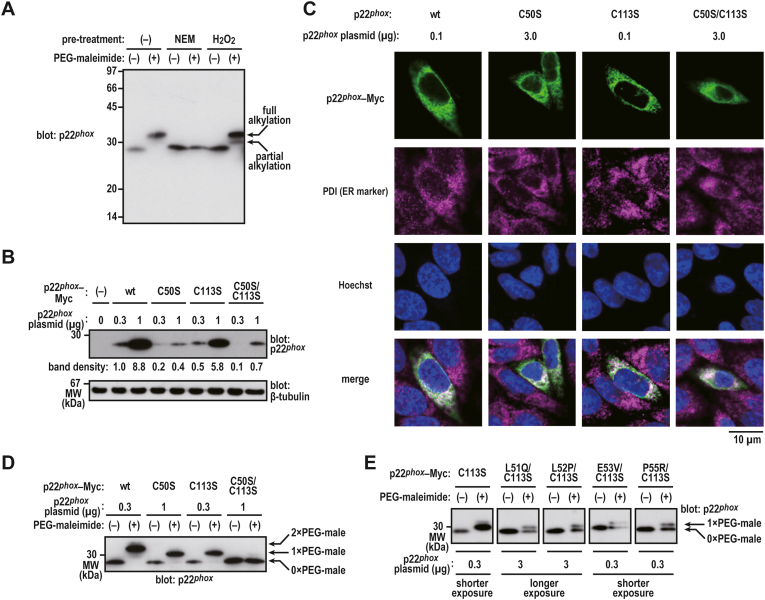
**Redox-sensitive cysteine in p22**^***phox***^ *A*, alkylation of p22^*phox*^. CHO–K1 cells (7 × 10^5^ cells in 6-well plates) were transfected with pcDNA3.1-wild-type (wt) p22^*phox*^–Myc (0.3 μg). Protein levels of exogenous p22^*phox*^–Myc in lysates were estimated via immunoblotting. *B*, expression levels of p22^*phox*^ mutant protein. CHO–K1 cells (7 × 10^5^ cells in 6-well plates) were transfected with the indicated plasmids: pcDNA3.1-wild-type (wt) p22^*phox*^–Myc (0.3 or 1 μg), pcDNA3.1-p22^*phox*^ (C50S)–Myc (0.3 or 1 μg), pcDNA3.1-p22^*phox*^ (C113S)–Myc (0.3 or 1 μg), or pcDNA3.1-p22^*phox*^ (C50S/C113S)–Myc (0.3 or 1 μg). The band densities were normalized to β-tubulin. *C*, distribution of exogenous mutant p22^*phox*^ in CHO–K1 cells. CHO–K1 cells (7 × 10^5^ cells in a 30-mm glass-bottom dish) were transfected with the indicated plasmids: pcDNA3.1-wild-type (wt) p22^*phox*^–Myc (0.1 μg), pcDNA3.1-p22^*phox*^ (C50S)–Myc (3 μg), pcDNA3.1-p22^*phox*^ (C113S)–Myc (0.1 μg), or pcDNA3.1-p22^*phox*^ (C50S/C113S)–Myc (3 μg). After fixation, the immunofluorescence signals were observed by confocal microscopy. Scale bars, 10 μm. *D and E*, alkylation of p22^*phox*^ mutant proteins. CHO–K1 cells (7 × 10^5^ cells in 6-well plates) were transfected with the indicated plasmids: pcDNA3.1-wild-type (wt) p22^*phox*^–Myc (0.3 μg), pcDNA3.1-p22^*phox*^ (C50S)–Myc (1 μg), pcDNA3.1-p22^*phox*^ (C113S)–Myc (0.3 μg), pcDNA3.1-p22^*phox*^ (C50S/C113S)–Myc (1 μg), pcDNA3.1-p22^*phox*^ (L51Q/C113S)–Myc (3 μg), pcDNA3.1-p22^*phox*^ (L52P/C113S)–Myc (3 μg), pcDNA3.1-p22^*phox*^ (E53V/C113S)–Myc (0.3 μg), or pcDNA3.1-p22^*phox*^ (P55R/C113S)–Myc (0.3 μg). 1 × PEG-mal, 1 × PEG-maleimide-modified p22^*phox*^; 2 × PEG-mal, 2 × PEG-maleimide-modified p22^*phox*^; 0 × PEG-mal, PEG-maleimide-unmodified p22^*phox*^. Protein levels of exogenous p22^*phox*^–Myc and endogenous β-tubulin (as loading control) in lysates were estimated via immunoblotting. Positions for marker proteins are indicated in kDa. Shorter exposure, shorter exposure films were used for scanning; longer exposure, longer exposure films were used for scanning. These experiments have been repeated more than three times with similar results.

Redox-sensitive cysteine residues in Nox2 subunits p67^*phox*^ and p47^*phox*^ participate in Nox2-based oxidase [[36], [37], [38], [39]]. Therefore, we focused on Cys-50, which is adjacent to the L^51^-L^52^-E^53^-P^55^ sequence (Fig. 1A). We determined whether the redox-sensitive cysteine residue(s) was/were present in the ER-retained p22^*phox*^ protein. CHO–K1 cells ectopically expressing p22^*phox*^–Myc were lysed in the presence or absence of methyl-PEG_24_-maleimide reagent [polyethylene glycol (PEG)-maleimide], which alkylates free thiol groups. PEG-maleimide-modified p22^*phox*^ was detected via immunoblotting (Fig. 4A). Following the pretreatment of cells with membrane permeable NEM, PEG-maleimide was observed to not react with the cysteines of p22^*phox*^ (Fig. 4A), suggesting that p22^*phox*^ possesses free thiol groups. When the cells are exposed to H_2_O_2_ as an oxidant, redox-sensitive cysteines are expected to be oxidized. We observed that the cysteines of p22^*phox*^ were partially alkylated by PEG-maleimide because of the oxidation of the free thiol group (Fig. 4A). These results suggest that p22^*phox*^ contains redox-sensitive cysteine residues.

As shown in Fig. 1A, p22^*phox*^ contains two cysteine residues in the amino acid sequence that corresponds to exons 3 and exon 5. To determine which cysteine residue(s) are responsible for the redox-sensitivity, we expressed mutant p22^*phox*^–Myc proteins harboring serine substitutions for Cys-50 and Cys-113 in CHO–K1 cells. As shown Fig. 4B, the C50S substitution resulted in a decrease in the amount of the mutant protein, suggesting that the mutation renders the protein unstable. In contrast, the substitution of Cys-113 exerted little effect on protein expression. The amount of p22^*phox*^ (C50S/C113S)–Myc double mutant protein was affected by the instability caused by the C50S substitution. These mutant proteins were colocalized with PDI (Fig. 4C), indicating that these mutant proteins were localized in the ER without the coexpression of Nox2. Next, the expression levels of each mutant protein were adjusted to be the same by varying the amount of plasmid used for transfection (Fig. 4D). When the transfected cells were lysed in the presence of PEG-maleimide, the apparent molecular masses of p22^*phox*^ (C50S)–Myc and p22^*phox*^ (C113S)–Myc were found to be slightly lower than that of wild-type p22^*phox*^–Myc (Fig. 4D). The apparent molecular masses of the double mutant proteins remained unchanged in the presence or absence of PEG-maleimide (Fig. 4D). These results indicate that Cys-50 and Cys-113 are redox-sensitive.

When the transfected cells were lysed in the presence of PEG-maleimide, only PEG-maleimide-modified p22^*phox*^ (C113S)–Myc was detected via immunoblotting (Fig. 4D). This result suggests that the Cys-50 of p22^*phox*^ was readily alkylated by PEG-maleimide. Interestingly, the Cys-50 of mutant p22^*phox*^ proteins carrying the L51Q/C113S, L52P/C113S, E53V/C113S, or P55R/C113S substitution was partially alkylated by PEG-maleimide (Fig. 4E). This result suggests that the exposure of the thiol in Cys-50, which is accessible to PEG-maleimide, might be affected by substitutions of amino acids adjacent to the Cys-50 of p22^*phox*^.

### 3.3 Role of redox-sensitive Cys-50 and Cys-113 in Nox activity

**Fig. 5:**
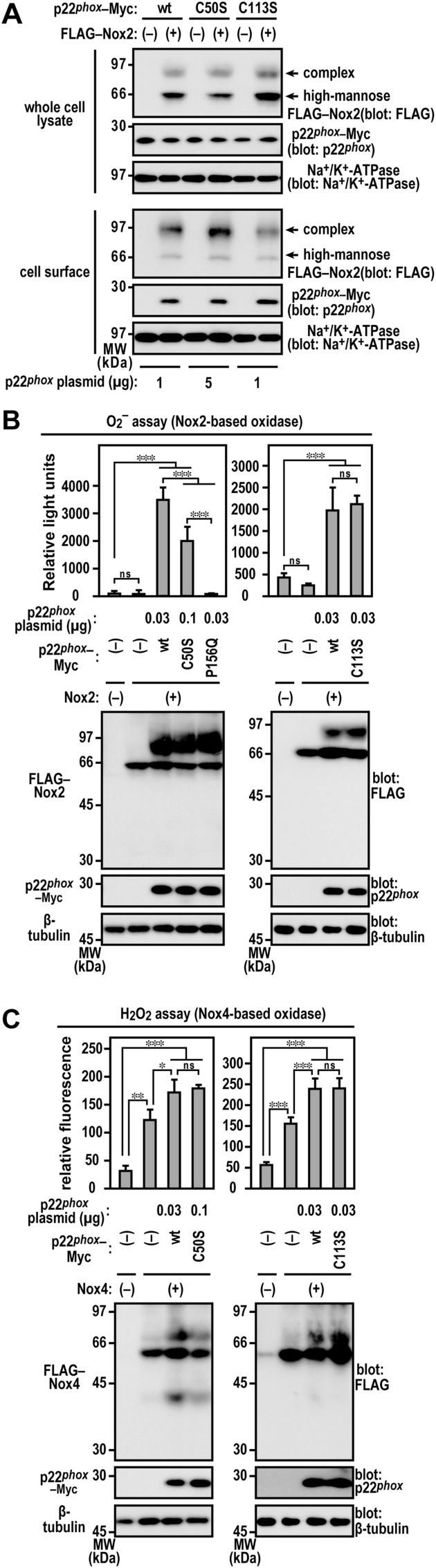
**Production of ROS by transfected CHO–K1 cells** *A*, cell surface localization of p22^*phox*^ with Nox2. CHO–K1 cells (3.2 × 10^6^ in a 9-cm dish) were transfected simultaneously with the indicated plasmids: pcDNA3.1-wild-type (wt) p22^*phox*^–Myc (1 μg), pcDNA3.1-p22^*phox*^ (C50S)–Myc (5 μg), pcDNA3.1-p22^*phox*^ (C113S)–Myc (1 μg), and/or pcDNA3.1-FLAG–Nox2 (9 μg). Complex, complex *N*-glycan-bearing Nox2; high-mannose, high mannose *N*-glycan-bearing Nox2. *B*, superoxide production by Nox2. CHO–K1 cells (7 × 10^5^ cells in 6-well plates) were transfected simultaneously with the indicated plasmids: pcDNA3.1-wild-type (wt) p22^*phox*^–Myc (0.03 μg), pcDNA3.1-p22^*phox*^ (C50S)–Myc (0.1 μg), pcDNA3.1-p22^*phox*^ (C113S)–Myc (0.03 μg), pcDNA3.1-p22^*phox*^ (P156Q)–Myc (0.03 μg), pcDNA3.1-FLAG–Nox2 (1 μg), pcDNA3.1-Myc–p67^*phox*^ (0.2 μg), and/or pcDNA3.1-Myc–p47^*phox*^ (0.2 μg). Superoxide production was assayed using superoxide dismutase inhibitable-chemiluminescence using Diogenes. Each graph represents the mean ± standard deviation of the chemiluminescence intensities integrated for 10 min after PMA stimulation from three independent transfections. *C*, H_2_O_2_ production by Nox4. CHO–K1 cells (7 × 10^5^ cells in 6-well plates) were transfected simultaneously with the indicated plasmids: pcDNA3.1-wild-type (wt) p22^*phox*^–Myc (0.03 μg), pcDNA3.1-p22^*phox*^ (C50S)–Myc (0.1 μg), or pcDNA3.1-p22^*phox*^ (C113S)–Myc (0.03 μg) and/or pcDNA3.1-FLAG–Nox4 (1 μg). H_2_O_2_ production was assayed using catalase-inhibitable fluorescence using the homovanillic acid–horseradish peroxidase detection system. Each graph represents the mean ± standard deviation of the fluorescence intensities, which were obtained from three independent transfections. Protein levels of the indicated proteins were estimated via immunoblotting. Positions for marker proteins are indicated in kDa. Statistical analysis was performed using Tukey–Kramer test. ***, *p* < 0.001; **, *p* < 0.01; *, *p* < 0.05; ns, no significance. These experiments have been repeated more than three times with similar results.

Next, we investigated the role of Cys-50 and Cys-113 in the ROS-generating activity of Nox2. Using a cell surface biotinylation assay, we demonstrated that p22^*phox*^ (C50S)–Nox2 and p22^*phox*^ (C113S)–Nox2 complexes localize at the plasma membrane (Fig. 5A). We expressed wild-type p22^*phox*^–Myc, p22^*phox*^ (C50S)–Myc, or p22^*phox*^ (C113S)–Myc together with a set of Nox2, p67^*phox*^, and p47^*phox*^. Under the same expression condition that was used for wild-type p22^*phox*^, p22^*phox*^ (P156Q)–Myc, which was defective in binding to p47^*phox*^ (a mutation found in a patient with CGD) [25,26], failed to support superoxide production by Nox2. In contrary, the production was sufficiently supported by the expression of p22^*phox*^ (C50S)–Myc and fully supported by that of p22^*phox*^ (C113S)–Myc (Fig. 5B). Nox4 also interacted with p22^*phox*^ to function as an H_2_O_2_-producing oxidase [35]. When FLAG–Nox4 and p22^*phox*^ mutant proteins were expressed, these mutant proteins activated Nox4 to the same extent as the wild-type p22^*phox*^ (Fig. 5C). These results indicate that the thiol groups of Cys-50 and Cys-113 are not required for the catalytic function of Nox.

### 3.4 Role of redox-sensitive Cys-50 in p22^phox^ protein stability

**Fig. 6:**
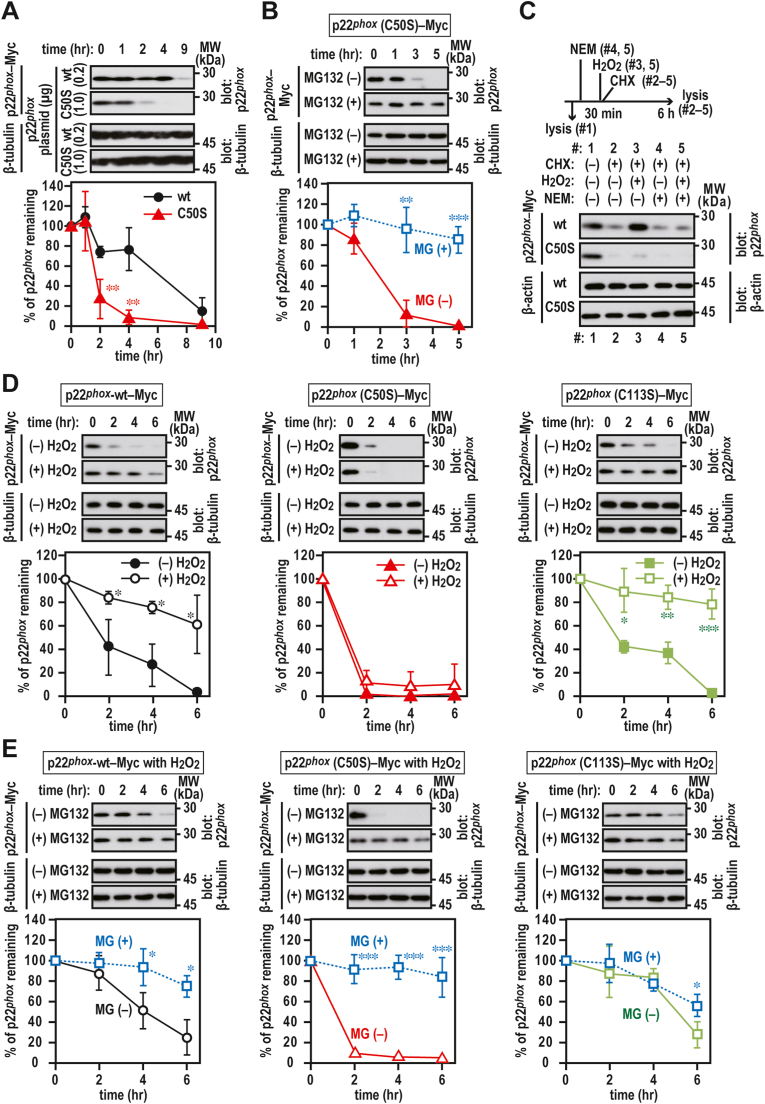
**Stabilization of p22**^***phox***^**by oxidation** *A and B*, stability of p22^*phox*^ mutant protein. CHO–K1 cells (7 × 10^5^ cells in 6-well plates) were transfected with the indicated plasmids: pcDNA3.1-wild-type (wt) p22^*phox*^–Myc (0.2 μg) or pcDNA3.1-p22^*phox*^ (C50S)–Myc (1.0 μg). The transfected cells were treated for 0, 1, 2, 4, 5 or 9 h with cycloheximide (CHX) in the presence or absence of 20 μM MG132. The graph represents the relative densities of the bands normalized to β-tubulin (n = 3). Statistical analysis was performed using Tukey–Kramer test. **, *p* < 0.01. *C*, effect of oxidation on p22^*phox*^ protein stability. CHO–K1 cells (7 × 10^5^ cells in 6-well plates) were transfected with the indicated plasmids: pcDNA3.1-wild-type (wt) p22^*phox*^–Myc (0.2 μg) or pcDNA3.1-p22^*phox*^ (C50S)–Myc (1.0 μg). The transfected cells were pretreated with 20 mM N-ethylmaleimide (NEM) for 30 min and then treated with (#5) or without (#4) 1 mM H_2_O_2_. In sample #3, the transfected cells were only treated with only 1 mM H_2_O_2_. In sample #1, the transfected cells were untreated. The pretreated cells (#2–5) were treated with 10 μg/ml cycloheximide (CHX) for 6 h. Protein levels of exogenous p22^*phox*^–Myc and endogenous β-tubulin (as loading control) were estimated via immunoblotting. *D and E*, stability of p22^*phox*^ mutant protein in the presence or absence of 1 mM H_2_O_2_. CHO–K1 cells (7 × 10^5^ cells in 6-well plates) were transfected with the indicated plasmids: pcDNA3.1-wild-type (wt) p22^*phox*^–Myc (0.2 μg), pcDNA3.1-p22^*phox*^ (C50S)–Myc (1.0 μg), or pcDNA3.1-p22^*phox*^ (C113S)–Myc (0.2 μg). The transfected cells were treated for 0, 2, 4, or 6 h with cycloheximide (CHX) in the presence or absence of 20 μM MG132. The graph represents the relative densities of the bands normalized to β-tubulin (n = 3). Protein levels of the indicated proteins were estimated via immunoblotting. Statistical analysis was performed using Tukey–Kramer test. ***, *p* < 0.001; **, *p* < 0.01; *, *p* < 0.05; ns, no significance. Positions for marker proteins are indicated in kDa. These experiments have been repeated more than three times with similar results.

The serine substitution of redox-sensitive Cys-50, but not Cys-113, affected the protein expression level (Fig. 4B). We investigated the effect of the serine substitution of Cys-50 on the stability of p22^*phox*^. As shown in Fig. 6A, the levels of p22^*phox*^ (C50S)–Myc mutant protein decreased to approximately 25% after exposure to CHX for 2 h. This result indicates that Cys-50 is indispensable for protein stability. The degradation of p22^*phox*^ (C50S)–Myc was considerably suppressed in the presence of MG132 (Fig. 6B), indicating that proteasome is responsible for the degradation of the p22^*phox*^ (C50S) mutant protein.

Furthermore, the oxidation of Cys-50 might affect p22^*phox*^ stabilization. To test this hypothesis, CHO–K1 cells expressing p22^*phox*^–Myc were treated with CHX in the presence or absence of H_2_O_2_ (Fig. 6C and D), the addition of which suppressed the degradation of wild-type p22^*phox*^–Myc and p22^*phox*^ (C113S)–Myc but not p22^*phox*^ (C50S)–Myc (Fig. 6D). The suppression effect was attenuated by pretreatment with NEM for blocking free thiols (Fig. 6C). The H_2_O_2_ treatment was able to partially inhibit the proteasome-dependent degradation of wild-type p22^*phox*^ and p22^*phox*^ (C113S) mutant protein (Fig. 6E). These results indicate that redox-sensitive Cys-50 is responsible for the stability of p22^*phox*^ protein in a thiol oxidation-dependent manner.

### 3.5 Role of Cys-50 of p22^phox^ in Nox2 and Nox4 protein stability

**Fig. 7:**
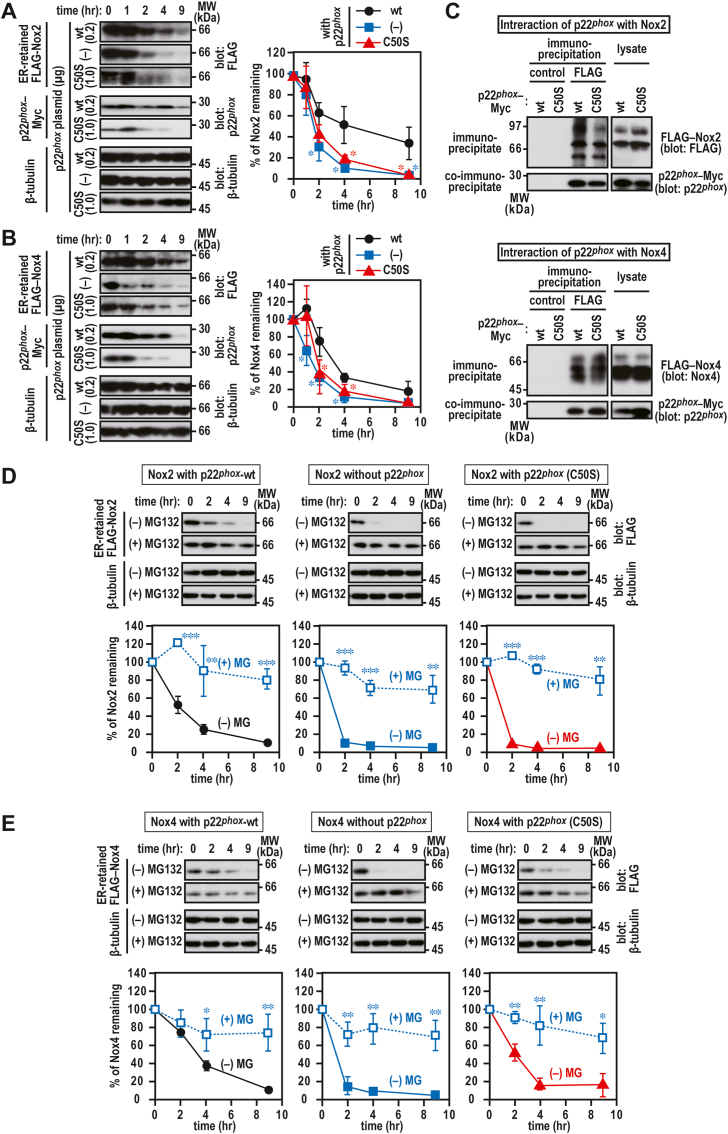
**Effect of p22**^***phox***^**mutant protein on the stability of ER-retained Nox2 or Nox4** *A, B, D, and E*, stability of Nox2 (*A and D*) and Nox4 (*B and E*) complexed with p22^*phox*^. CHO–K1 cells (7 × 10^5^ cells in 6-well plates) were simultaneously transfected with the indicated plasmids: cDNA3.1-wild-type (wt) p22^*phox*^–Myc (0.1 μg) or pcDNA3.1-p22^*phox*^ (C50S)–Myc (0.3 μg) and pcDNA3.1-FLAG–Nox2 (1.0 μg) or pcDNA3.1-FLAG–Nox4 (1.0 μg). The transfected cells were exposed to 10 μg/ml Brefeldin A for 1 h and then with treated for 0, 1, 2, 4, or 9 h with cycloheximide (CHX) in the presence or absence of 20 μM MG132. Each graph represents the relative densities of the bands normalized to β-tubulin (n = 3). Statistical analysis was performed using Tukey–Kramer test. ***, *p* < 0.001; **, *p* < 0.01; *, *p* < 0.05. *C*, interaction of p22^*phox*^ with Nox2 and Nox4. CHO–K1 cells (2.1 × 10^6^ in a 6-cm dish) were transfected simultaneously with the indicated plasmids: pcDNA3.1-wild-type (wt) p22^*phox*^–Myc (0.1 μg) or pcDNA3.1-p22^*phox*^ (C50S)–Myc (3.0 μg) and pcDNA3.1-FLAG–Nox2 (3.0 μg) or pcDNA3.1-FLAG–Nox4 (3.0 μg). Protein levels of the indicated proteins were estimated via immunoblotting. Positions for marker proteins are indicated in kDa. The data are representative of results from three independent experiments. control, Mouse IgG–Agarose; FLAG, ANTI-FLAG® M2 Agarose Affinity Gel. These experiments have been repeated more than three times with similar results.

We investigated the effect of p22^*phox*^ (C50S)–Myc on the stability of Nox2 and Nox4. The coexpression of wild-type p22^*phox*^–Myc significantly stabilized ER-localized Nox2 (Fig. 7A) and Nox4 (Fig. 7B) carrying high-mannose glycan proteins. In contrast, these effects were not observed with p22^*phox*^ (C50S)–Myc. Nox2 and Nox4 interacted with p22^*phox*^ (C50S)–Myc as well as with wild-type p22^*phox*^–Myc (Fig. 7C). Degradation of Nox2 and Nox4 was considerably suppressed in the presence of MG132 (Fig. 7D and E), indicating that proteasome is responsible for degradation of Nox2 and Nox4 complexed with p22^*phox*^. These results indicate that the instability of p22^*phox*^ (C50S) protein affects the stability of Nox2 and Nox4 proteins when complexed with p22^*phox*^.

### 3.6 Recognition of p22^phox^ mutant proteins by Derlin-1

**Fig. 8:**
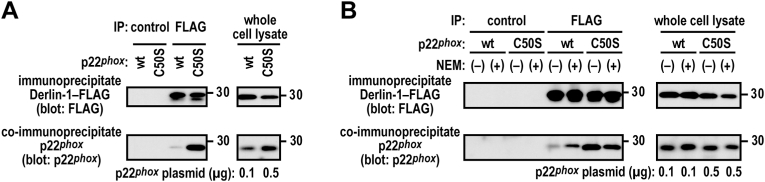
**Role of Cys-50 in binding of p22**^***phox***^**to Derlin-1** *A*, interaction of Derlin-1 with p22^*phox*^ mutant proteins. CHO–K1 cells (2.1 × 10^6^ in a 6-cm dish) were transfected simultaneously with the indicated plasmids: pcDNA3.1-Derlin-1–FLAG (0.5 μg) and pcDNA3.1-wild-type (wt) p22^*phox*^–Myc (0.1 μg or 0.5 μg) or pcDNA3.1-p22^*phox*^ (C50S)–Myc (0.5 μg). control, Mouse IgG–Agarose; FLAG, ANTI-FLAG® M2 Agarose Affinity Gel. *B*, effect of alkylation on interaction between Derlin-1 and p22^*phox*^. CHO–K1 cells (2.1 × 10^6^ in a 6-cm dish) were transfected simultaneously with the indicated plasmids: pcDNA3.1-Derlin-1–FLAG (0.5 μg) and pcDNA3.1-wild-type (wt) p22^*phox*^–Myc (0.1 μg) or pcDNA3.1-p22^*phox*^ (C50S)–Myc (0.5 μg). The transfected cells were pretreated with 20 mM N-ethylmaleimide (NEM) for 10 min control, Mouse IgG–Agarose; FLAG, ANTI-FLAG® M2 Agarose Affinity Gel. Protein levels of the indicated proteins were estimated via immunoblotting. Positions for marker proteins are indicated in kDa. The data are representative of results from three independent experiments.

Derlin-1 is implicated in ERAD and interacts with misfolded transmembrane proteins during their transfer from the ER to the cytosolic proteasome [[27], [28], [29], [30]]. To determine whether p22^*phox*^ mutant proteins bind to Derlin-1, Derlin-1–FLAG proteins were immunoprecipitated from the cell lysates of CHO–K1 cells expressing exogenous p22^*phox*^–Myc and Derlin-1–FLAG proteins. In the whole cell lysate, p22^*phox*^ (C50S)–Myc was observed at an extent similar to that of wild-type p22^*phox*^–Myc at a plasmid ratio of 1:5 (wild-type:C50S); however, Derlin-1 coprecipitated p22^*phox*^ (C50S)–Myc more efficiently than the wild-type p22^*phox*^–Myc (Fig. 8A). Pretreatment with NEM for blocking free thiols in wild-type p22^*phox*^–Myc promoted the interaction of Derlin-1–FLAG with wild-type p22^*phox*^–Myc (Fig. 8B). However, the NEM pretreatment did not enhance Derlin-1 binding to p22^*phox*^ (C50S)–Myc. These results indicate that redox-sensitive Cys-50 of p22^*phox*^ is responsible for recognition by Derlin-1.

**Fig. 9:**
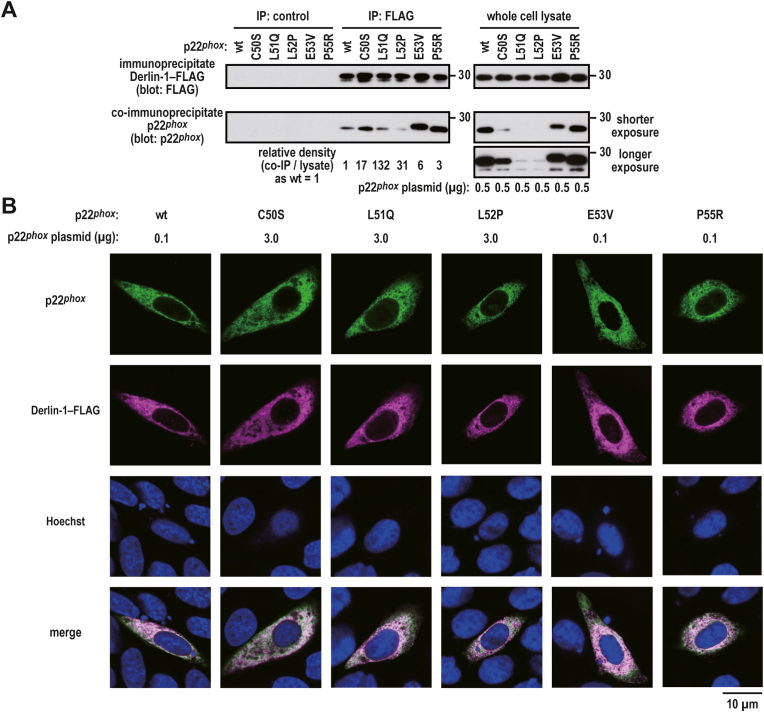
**Binding of p22**^***phox***^**mutant proteins to Derlin-1** *A*, interaction of Derlin-1 with p22^*phox*^ mutant proteins. CHO–K1 cells (2.1 × 10^6^ in a 6-cm dish) were transfected simultaneously with the indicated plasmids: pcDNA3.1-Derlin-1–FLAG (0.5 μg) and pcDNA3.1-wild-type (wt) p22^*phox*^–Myc (0.5 μg), pcDNA3.1-p22^*phox*^ (C50S)–Myc (0.5 μg), pcDNA3.1-p22^*phox*^ (L51Q)–Myc (0.5 μg), pcDNA3.1-p22^*phox*^ (L52P)–Myc (0.5 μg), pcDNA3.1-p22^*phox*^ (E53V)–Myc (0.5 μg), or pcDNA3.1-p22^*phox*^ (P55R)–Myc (0.5 μg). control, Mouse IgG–Agarose; FLAG, ANTI-FLAG® M2 Agarose Affinity Gel. The protein levels of the indicated proteins were estimated via immunoblotting. The positions for marker proteins are indicated in kDa. Shorter exposure, shorter exposure films were used for scanning; longer exposure, longer exposure films were used for scanning. *B*, distribution of exogenous p22^*phox*^ and Derlin-1 in CHO–K1 cells. CHO–K1 cells (2.1 × 10^6^ in a 6-cm dish) were transfected simultaneously with the indicated plasmids: pcDNA3.1-Derlin-1–FLAG (0.1 μg) and pcDNA3.1-wild-type (wt) p22^*phox*^–Myc (0.1 μg), pcDNA3.1-p22^*phox*^ (C50S)–Myc (3 μg), pcDNA3.1-p22^*phox*^ (L51Q)–Myc (3 μg), pcDNA3.1-p22^*phox*^ (L52P)–Myc (3 μg), pcDNA3.1-p22^*phox*^ (E53V)–Myc (0.1 μg), or pcDNA3.1-p22^*phox*^ (P55R)–Myc (0.1 μg). After fixation, the immunofluorescence signals were observed by confocal microscopy. Scale bars, 10 μm. The data are representative of results from three independent experiments.

**Fig. 10:**
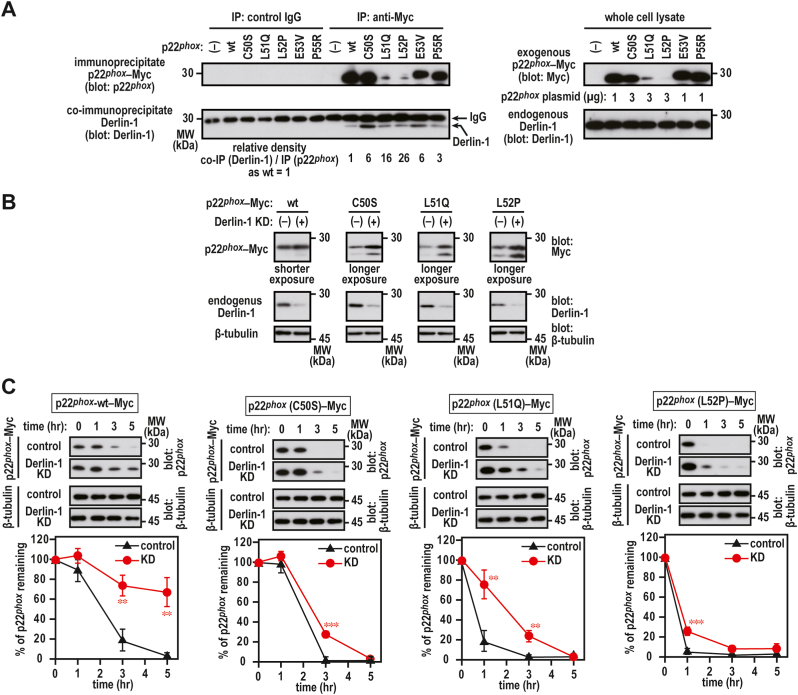
**Effect of Derlin-1 knockdown on the stability of p22**^***phox***^**in HeLa cells** *A*, interaction of endogenous Derlin-1 with p22^*phox*^ mutant proteins. HeLa cells (2.1 × 10^6^ in a 6-cm dish) were transfected simultaneously with the indicated plasmids: pcDNA3.1-wild-type (wt) p22^*phox*^–Myc (1 μg), pcDNA3.1-p22^*phox*^ (C50S)–Myc (3 μg), pcDNA3.1-p22^*phox*^ (L51Q)–Myc (3 μg), pcDNA3.1-p22^*phox*^ (L52P)–Myc (3 μg), pcDNA3.1-p22^*phox*^ (E53V)–Myc (1 μg), or pcDNA3.1-p22^*phox*^ (P55R)–Myc (1 μg). control IgG, anti-mouse control IgG antibody; anti Myc, anti-Myc antibody (9E10). Protein levels of the indicated proteins were estimated via immunoblotting. *B*, effect of Derlin-1 knockdown on the expression levels of p22^*phox*^ mutant proteins. HeLa cells (2.1 × 10^6^ in a 6-cm dish) were transfected with the indicated plasmids: pcDNA3.1-wild-type (wt) p22^*phox*^–Myc (0.1 μg), pcDNA3.1-p22^*phox*^ (C50S)–Myc (3 μg), pcDNA3.1-p22^*phox*^ (L51Q)–Myc (3 μg), or pcDNA3.1-p22^*phox*^ (L52P)–Myc (3 μg). Protein levels of the indicated proteins were estimated via immunoblotting. Shorter exposure, shorter exposure films were used for scanning; longer exposure, longer exposure films were used for scanning. *C*, effect of Derlin-1 knockdown on the stability of p22^*phox*^ mutant protein. HeLa cells (2.1 × 10^6^ in a 6-cm dish) were transfected with the indicated plasmids: pcDNA3.1-wild-type (wt) p22^*phox*^–Myc (0.1 μg), pcDNA3.1-p22^*phox*^ (C50S)–Myc (3 μg), pcDNA3.1-p22^*phox*^ (L51Q)–Myc (3 μg), or pcDNA3.1-p22^*phox*^ (L52P)–Myc (3 μg). The transfected cells were treated for 0, 1, 3, or 5 h with cycloheximide (CHX). Protein levels of exogenous p22^*phox*^–Myc and endogenous β-tubulin (as loading control) were estimated via immunoblotting. Statistical analysis was performed using Tukey–Kramer test. ***, *p* < 0.001; **. Positions for marker proteins are indicated in kDa. These experiments have been repeated more than three times with similar results.

In whole cell lysates, p22^*phox*^ (E53V)–Myc and p22^*phox*^ (P55R)–Myc were observed at an extent similar to that of wild-type p22^*phox*^–Myc at a plasmid ratio of 1:1 (wild-type:mutant proteins), and Derlin-1 was strongly bound to these mutant proteins (Fig. 9A). Although the expression levels of p22^*phox*^ (L51Q)–Myc and p22^*phox*^ (L52P)–Myc were considerably lower than those of wild-type p22^*phox*^, Derlin-1 coprecipitated these mutant proteins at an extent similar to that of wild-type p22^*phox*^–Myc (Fig. 9A). These mutant proteins were observed to be colocalized with Derlin-1–FLAG via a confocal laser microscope (Fig. 9B). These results suggest that Derlin-1 participates in ERAD-mediated degradation of p22^*phox*^. To test this possibility, we attempted to knock down Derlin-1 in HeLa cells using commercially available and validated siRNA against human Derlin-1. We observed that endogenous Derlin-1 was efficiently coprecipitated with the anti-Myc antibody but not control IgG from the cell lysates of the cells expressing exogenouse p22^*phox*^–Myc mutant proteins (Fig. 10A). Derlin-1 knockdown partially restored the expression levels of p22^*phox*^ mutant proteins (Fig. 10B), which were markedly underexpressed compared with the wild-type protein (Fig. 2, Fig. 4B). Derlin-1 depletion partially suppressed the degradation of p22^*phox*^ mutant proteins (Fig. 10C). These results suggest that p22^*phox*^ mutant proteins are recognized by Derlin-1 for proteasomal degradation.

## 4 Discussion

**Fig. 11:**
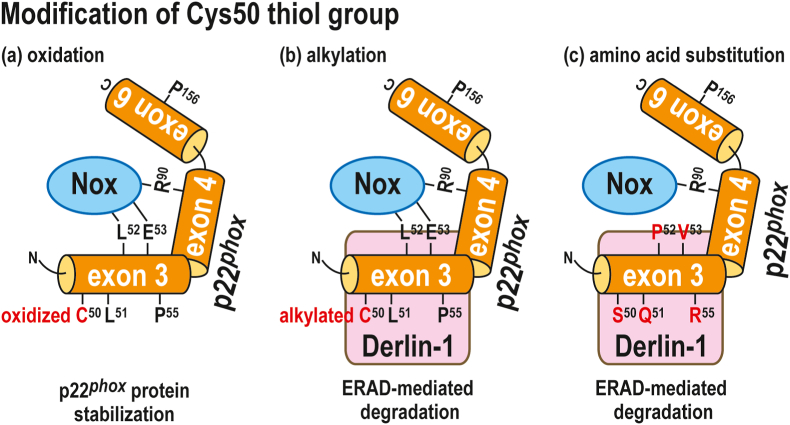
**Schematic representation of the p22**^***phox***^**degradation through ERAD system** Regulation of Derlin-1-mediated degradation of p22^*phox*^ by thiol modification. The oxidation of the Cys-50 thiol group by H_2_O_2_ enhanced the stability of ER-retained p22^*phox*^ protein (a). The thiol on Cys-50 by alkylation resulted in p22^*phox*^ degradation through the ERAD pathway (b). C50S substitution resulted in p22^*phox*^ degradation through the ERAD pathway (c).

In the present study, we demonstrated that Leu-51, Leu-52, Glu-53, and Pro-55 in the amino acid sequence that corresponds to exon 3 are responsible for p22^*phox*^ protein stability. In addition, the serine substitution of Cys-50, which is adjacent to the L^51^-L^52^-E^53^-P^55^ sequence and is redox-sensitive, leads to protein instability. This instability affects the stability of Nox2 and Nox4 when complexed with p22^*phox*^. Furthermore, blocking the free thiol of Cys-50 using alkylating agents or the serine substitution of Cys-50 promotes the association of p22^*phox*^ with Derlin-1, a key component of the ERAD system. In addition, L51Q, L52P, E53V, and P55R mutant proteins bind to Derlin-1 more efficiently than the wild-type protein. These findings suggest that the C-terminal region adjacent to Cys-50 (amino acids 50–55, including Cys-50) is responsible for p22^*phox*^ protein stability (Fig. 11).

Missense mutations in the *CYBA* exon 3 resulted in p22^*phox*^ protein becoming undetectable [16]. We characterized the p22^*phox*^ mutant proteins (L51Q, L52P, E53V, and P55R), which were unstable (Figs. 1A and 2). Because Nox2 protein stability is dependent on the formation of a complex with p22^*phox*^ in the ER [10,13,14], p22^*phox*^ deficiency resulting from the substitution of amino acids leads to reduced protein expression level of the main Nox2 subunit of phagocyte NADPH oxidase, consequently causing CGD. Considering that a mutational hotspot is located in the *CYBA* exon 3 of patients with A22° type CGD, the amino acid sequence (residues 44–68) that corresponds to exon 3 appears to be responsible for p22^*phox*^ protein stability.

In a previous study [40], performed screening of a library of peptides spanning the amino acid sequence of p22^*phox*^ for the inhibition of Nox2 activity. These peptides interfere with the binding of p47^*phox*^ to Nox2–p22^*phox*^ [40]: p47^*phox*^ primarily binds to a proline-rich region (residues 151–160) in the C-terminal cytosolic tail of p22^*phox*^. Furthermore, the screening revealed that amino acid residues 47–61 are responsible for Nox2 activity [40]. These peptides may promote the dissociation of p22^*phox*^ from the Nox2–p22^*phox*^ complex. In addition, the N-terminal region of p22^*phox*^ is required for Nox2 protein maturation [41], which completely depends on binding to p22^*phox*^. In the present study, we demonstrated that p22^*phox*^ (L52P) and p22^*phox*^ (E53V) are defective in binding to Nox2 (Fig. 3A). Therefore, residues 44–68 that correspond to exon 3 may be the region responsible for binding to Nox2.

A previous study showed that p22^*phox*^ is a target for ubiquitination and treatment with proteasome inhibitors suppresses p22^*phox*^ degradation [42]. The ERAD system promotes the translocation of misfolded proteins from the ER to the cytosol [43]. They are then degraded by the ubiquitin–proteasome system [43]. Derlin-1 is part of a channel for retro-translocation and is essential for the degradation of misfolded membrane proteins [[27], [28], [29], [30]], such as cystic fibrosis transmembrane conductance regulator (CFTR)-ΔF508 mutant protein [44,45]. Herein, we demonstrated that in the ER, the substitution of Leu-51, Leu-52, Glu-53, and Pro-55 facilitates the interaction of p22^*phox*^ with Derlin-1 (Fig. 8, Fig. 9, Fig. 10). Thus, L51Q, L52P, E53V, and P55R mutant proteins in patients with A22° type CGD are degraded through the ERAD pathway (Fig. 11).

The L52P and E53V mutations impair the binding of p22^*phox*^ to Nox2 (Fig. 3). As the p22^*phox*^ protein stability depends on the complex formation with Nox2 [46], monomer p22^*phox*^ might be degraded in the phagocytes through the ERAD pathway, resulting in the A22° type of CGD. However, L51Q and P55R mutant proteins bind Nox2, although they are unstable. It is currently unknown whether p22^*phox*^ would be degraded in phagocytes before binding to *de novo* Nox2 or after complex formation with Nox2.

Blocking the thiol on Cys-50 by alkylation or substituting it with hydroxyl group resulted in p22^*phox*^ degradation through the ERAD pathway (Fig. 6). In contrast, the oxidation of the Cys-50 thiol group by H_2_O_2_ enhanced the stability of ER-retained p22^*phox*^ protein (Fig. 6) and blocked the alkylation of thiols (Fig. 4). In addition, the degradation of the Nox2–p22^*phox*^ and Nox4–p22^*phox*^ complexes were accelerated by the serine substitution of the redox-sensitive Cys-50 (Fig. 7). Hence, the protein expression of Nox2 and Nox4 might be regulated by the modification of the Cys-50 thiol group (Fig. 11). The effects of Cys-50 modification on the stability of p22^*phox*^ appear to be important for Nox4-based oxidase activity. Nox2, which is heterodimerized with p22^*phox*^, is activated depending on complex formation with cytosolic activating proteins and Rac in response to cell stimulation. Thus, the switch for activating Nox2 is turned on or off by the formation or dissociation of the complex. In contrast, Nox4, which is heterodimerized with p22^*phox*^, constitutively produces ROS in a cytosolic activating protein-independent manner. Because the switch for Nox4 activity cannot be easily turned off, Nox4 degradation appears to be an effective way to turn off Nox4 activity. Nox4 protein stability is dependent on the presence of p22^*phox*^ (Fig. 7B) [22,23]. In addition, Nox4 primarily localizes in the ER [21,47,48]. Thus, we propose that the modification of the Cys-50 thiol group results in the degradation of p22^*phox*^ through the ERAD pathway and is a switch for Nox4 inactivation.

Misfolded CFTR-ΔF508 membrane protein can escape ERAD through low-temperature treatment [49] or chemical (VX-809) treatment [50]. These treatments rescue CFTR-ΔF508 trafficking from the ER to the plasma membrane and partially restore the function of the chloride channel. Because p22^*phox*^ (L51Q) and p22^*phox*^ (P55R) retain the ability to associate with Nox2, the strategy of the escape of mutant proteins from the ERAD system may overcome defective Nox2-based activity in patients with A22° type CGD. Modification of the thiol present in Cys-50, which is adjacent to the L^51^-L^52^-E^53^-P^55^ sequence in the amino acid sequence that corresponds to exon 3, is responsible for both avoiding and promoting the degradation of p22^*phox*^. Thus, the identification of molecules involved in the modification of Cys-50 thiol may be valuable for future studies. Molecules that bind/dissociate depending on modification may also be discovered. Additionally, in the present study, we used the indirect method of detecting cysteine oxidation based on reactivity loss with thiol-modifying reagents in the cell lysate. Proteomic approaches are proposed by the Chouchani [51] and Carroll [52] groups for the characterization of cysteine thiol modifications. Using their proposed methods in the future, we would identify post-translational p22phox cysteine residue modifications in intact primary phagocytes.

## Author contributions

Conceptualization: KM. Investigation: KM, SO, and CK. Project administration: KM. Visualization: KM. Writing – original draft: KM. Writing – review & editing: KM, SO, MK, TK, AY, and FK.

## Funding and additional information

This study was supported in part by JSPS KAKENHI [grant number JP17K08637 (KM), JP22K06934 (KM)], the 10.13039/100016189Wesco Scientific Promotion Foundation (KM), the 10.13039/100007449Takeda Science Foundation (KM), the Okayama Medical Foundation (KM), Teraoka Scholarship Foundation (KM) and Research Project Grants [nos. R01S-003 (KM)] from Kawasaki Medical School.

## Declaration of competing interest

The authors declare that they have no conflicts of interest.

## Data Availability

Data will be made available on request.

## References

[1] Nauseef W.M. (2019). The phagocyte NOX2 NADPH oxidase in microbial killing and cell signaling. Curr. Opin. Immunol..

[2] Stasia M.J. (2016). CYBA encoding p22(phox), the cytochrome b558 alpha polypeptide: gene structure, expression, role and physiopathology. Gene.

[3] Brandes R.P., Weissmann N., Schröder K. (2014). Nox family NADPH oxidases: molecular mechanisms of activation. Free Radic. Biol. Med..

[4] Lambeth J.D., Neish A.S. (2014). Nox enzymes and new thinking on reactive oxygen: a double-edged sword revisited. Annu. Rev. Pathol..

[5] Leto T.L., Morand S., Hurt D., Ueyama T. (2009). Targeting and regulation of reactive oxygen species generation by Nox family NADPH oxidases. Antioxidants Redox Signal..

[6] Schröder K., Weissmann N., Brandes R.P. (2017). Organizers and activators: cytosolic Nox proteins impacting on vascular function. Free Radic. Biol. Med..

[7] Rousset F., Carnesecchi S., Senn P., Krause K.H. (2015). NOX3-TARGETED therapies for inner ear pathologies. Curr. Pharmaceut. Des..

[8] Sirokmány G., Donkó Á., Geiszt M. (2016). Nox/Duox family of NADPH oxidases: lessons from knockout mouse models. Trends Pharmacol. Sci..

[9] Roos D. (2016). Chronic granulomatous disease. Br. Med. Bull..

[10] DeLeo F.R., Burritt J.B., Yu L., Jesaitis A.J., Dinauer M.C., Nauseef W.M. (2000). Processing and maturation of flavocytochrome *b*_558_ include incorporation of heme as a prerequisite for heterodimer assembly. J. Biol. Chem..

[11] Yu L., DeLeo F.R., Biberstine-Kinkade K.J., Renee J., Nauseef W.M., Dinauer M.C. (1999). Biosynthesis of flavocytochrome *b*_558_. p91^*phox*^ is synthesized as a 65-kDa precursor. J. Biol. Chem..

[12] Porter C.D., Parkar M.H., Verhoeven A.J., Levinsky R.J., Collins M.K., Kinnon C. (1994). p22-*phox*-deficient chronic granulomatous disease: reconstitution by retrovirus-mediated expression and identification of a biosynthetic intermediate of gp91-*phox*. Blood.

[13] Zhen L., King A.A., Xiao Y., Chanock S.J., Orkin S.H., Dinauer M.C. (1993). Gene targeting of X chromosome-linked chronic granulomatous disease locus in a human myeloid leukemia cell line and rescue by expression of recombinant gp91^*phox*^. Proc. Natl. Acad. Sci. U. S. A..

[14] Parkos C.A., Dinauer M.C., Jesaitis A.J., Orkin S.H., Curnutte J.T. (1989). Absence of both the 91kD and 22kD subunits of human neutrophil cytochrome b in two genetic forms of chronic granulomatous disease. Blood.

[15] Weening R.S., Corbeel L., de Boer M., Lutter R., van Zwieten R., Hamers M.N., Roos D. (1985). Cytochrome b deficiency in an autosomal form of chronic granulomatous disease. A third form of chronic granulomatous disease recognized by monocyte hybridization. J. Clin. Invest..

[16] Roos D., van Leeuwen K., Hsu A.P., Priel D.L., Begtrup A., Brandon R., Rawat A., Vignesh P., Madkaikar M., Stasia M.J. (2021). Hematologically important mutations: the autosomal forms of chronic granulomatous disease (third update). Blood Cells Mol. Dis..

[17] de Boer M., de Klein A., Hossle J.P., Seger R., Corbeel L., Weening R.S., Roos D. (1992). Cytochrome *b*_558_-negative, autosomal recessive chronic granulomatous disease: two new mutations in the cytochrome *b*_558_ light chain of the NADPH oxidase (p22-*phox*). Am. J. Hum. Genet..

[18] Kuroda J., Nakagawa K., Yamasaki T., Nakamura K., Takeya R., Kuribayashi F., Imajoh-Ohmi S., Igarashi K., Shibata Y., Sueishi K. (2005). The superoxide-producing NAD(P)H oxidase Nox4 in the nucleus of human vascular endothelial cells. Gene Cell..

[19] Kim Y.W., Byzova T.V. (2014). Oxidative stress in angiogenesis and vascular disease. Blood.

[20] von Löhneysen K., Noack D., Wood M.R., Friedman J.S., Knaus U.G. (2010). Structural insights into Nox4 and Nox2: motifs involved in function and cellular localization. Mol. Cell Biol..

[21] Miyano K., Okamoto S., Yamauchi A., Kawai C., Kajikawa M., Kiyohara T., Tamura M., Taura M., Kuribayashi F. (2020). The NADPH oxidase NOX4 promotes the directed migration of endothelial cells by stabilizing vascular endothelial growth factor receptor 2 protein. J. Biol. Chem..

[22] Zana M., Péterfi Z., Kovács H.A., Tóth Z.E., Enyedi B., Morel F., Paclet M.H., Donkó Á., Morand S., Leto T.L. (2018). Interaction between p22^*phox*^ and Nox4 in the endoplasmic reticulum suggests a unique mechanism of NADPH oxidase complex formation. Free Radic. Biol. Med..

[23] Miyano K., Okamoto S., Yamauchi A., Kawai C., Kajikawa M., Kiyohara T., Itsumi M., Taura M., Kuribayashi F. (2022). The downregulation of NADPH oxidase Nox4 during hypoxia in hemangioendothelioma cells: a possible role of p22^*phox*^ on Nox4 protein stability. Free Radic. Res..

[24] Nakano Y., Longo-Guess C.M., Bergstrom D.E., Nauseef W.M., Jones S.M., Bánfi B. (2008). Mutation of the Cyba gene encoding p22phox causes vestibular and immune defects in mice. J. Clin. Invest..

[25] Sumimoto H., Kage Y., Nunoi H., Sasaki H., Nose T., Fukumaki Y., Ohno M., Minakami S., Takeshige K. (1994). Role of Src homology 3 domains in assembly and activation of the phagocyte NADPH oxidase. Proc. Natl. Acad. Sci. U. S. A..

[26] Leto T.L., Adams A.G., de Mendez I. (1994). Assembly of the phagocyte NADPH oxidase: binding of Src homology 3 domains to proline-rich targets. Proc. Natl. Acad. Sci. U. S. A..

[27] Lilley B.N., Ploegh H.L. (2004). A membrane protein required for dislocation of misfolded proteins from the ER. Nature.

[28] Ye Y., Shibata Y., Yun C., Ron D., Rapoport T.A. (2004). A membrane protein complex mediates retro-translocation from the ER lumen into the cytosol. Nature.

[29] Sugiyama T., Murao N., Kadowaki H., Takao K., Miyakawa T., Matsushita Y., Katagiri T., Futatsugi A., Shinmyo Y., Kawasaki H., Sakai J., Shiomi K., Nakazato M., Takeda K., Mikoshiba K., Ploegh H.L., Ichijo H., Nishitoh H. (2021). ERAD components Derlin-1 and Derlin-2 are essential for postnatal brain development and motor function. iScience.

[30] Nejatfard A., Wauer N., Bhaduri S., Conn A., Gourkanti S., Singh N., Kuo T., Kandel R., Amaro R.E., Neal S.E. (2021). Derlin rhomboid pseudoproteases employ substrate engagement and lipid distortion to enable the retrotranslocation of ERAD membrane substrates. Cell Rep..

[31] Miyano K., Okamoto S., Yamauchi A., Kajikawa M., Kiyohara T., Taura M., Kawai C., Kuribayashi F. (2020). Constitutive activity of NADPH oxidase 1 (Nox1) that promotes its own activity suppresses the colon epithelial cell migration. Free Radic. Res..

[32] Kawai C., Yamauchi A., Kuribayashi F. (2018). Monoclonal antibody 7D5 recognizes the R147 epitope on the gp91^*phox*^, phagocyte flavocytochrome *b*_558_ large subunit. Microbiol. Immunol..

[33] Miyano K., Okamoto S., Kajikawa M., Kawai C., Kanagawa T., Tominaga S., Yamauchi A., Kuribayashi F. (2021). The efficient detection of membrane protein with immunoblotting: lessons from cold-temperature denaturation. Kawasaki Med. J..

[34] Biberstine-Kinkade K.J., Yu L., Stull N., LeRoy B., Bennett S., Cross A., Dinauer M.C. (2002). Mutagenesis of p22^*phox*^ histidine 94. A histidine in this position is not required for flavocytochrome *b*_558_ function. J. Biol. Chem..

[35] von Löhneysen K., Noack D., Jesaitis A.J., Dinauer M.C., Knaus U.G. (2008). Mutational analysis reveals distinct features of the Nox4-p22^*phox*^ complex. J. Biol. Chem..

[36] Gimenez M., Veríssimo-Filho S., Wittig I., Schickling B.M., Hahner F., Schürmann C., Netto L.E.S., Rosa J.C., Brandes R.P., Sartoretto S., De Lucca Camargo L., Abdulkader F., Miller F.J., Lopes L.R. (2019). Redox activation of Nox1 (NADPH oxidase 1) involves an intermolecular disulfide bond between protein disulfide isomerase and p47phox in vascular smooth muscle cells. Arterioscler. Thromb. Vasc. Biol..

[37] Fradin T., Bechor E., Berdichevsky Y., Dahan I., Pick E. (2018). Binding of p67phox to Nox2 is stabilized by disulfide bonds between cysteines in the 369 Cys-Gly-Cys371 triad in Nox2 and in p67phox. J. Leukoc. Biol..

[38] Dahan I., Smith S.M., Pick E. (2015). A Cys-Gly-Cys triad in the dehydrogenase region of Nox2 plays a key role in the interaction with p67phox. J. Leukoc. Biol..

[39] de A Paes A.M., Veríssimo-Filho S., Guimarães L.L., Silva A.C., Takiuti J.T., Santos C.X., Janiszewski M., Laurindo F.R., Lopes L.R. (2011). Protein disulfide isomerase redox-dependent association with p47(phox): evidence for an organizer role in leukocyte NADPH oxidase activation. J. Leukoc. Biol..

[40] Dahan I., Issaeva I., Gorzalczany Y., Sigal N., Hirshberg M., Pick E. (2002). Mapping of functional domains in the p22^*phox*^ subunit of flavocytochrome *b*_559_ participating in the assembly of the NADPH oxidase complex by “peptide walking. J. Biol. Chem..

[41] Zhu Y., Marchal C.C., Casbon A.J., Stull N., von Löhneysen K., Knaus U.G., Jesaitis A.J., McCormick S., Nauseef W.M., Dinauer M.C. (2006). Deletion mutagenesis of p22^*phox*^ subunit of flavocytochrome *b*_558_: identification of regions critical for gp91phox maturation and NADPH oxidase activity. J. Biol. Chem..

[42] Block K., Gorin Y., Hoover P., Williams P., Chelmicki T., Clark R.A., Yoneda T., Abboud H.E. (2007). NAD(P)H oxidases regulate HIF-2α protein expression. J. Biol. Chem..

[43] Ruggiano A., Foresti O., Carvalho P. (2014). Quality control: ER-associated degradation: protein quality control and beyond. J. Cell Biol..

[44] Younger J.M., Chen L., Ren H.Y., Rosser M.F., Turnbull E.L., Fan C.Y., Patterson C., Cyr D.M. (2006). Sequential quality-control checkpoints triage misfolded cystic fibrosis transmembrane conductance regulator. Cell.

[45] Sun F., Zhang R., Gong X., Geng X., Drain P.F., Frizzell R.A. (2006). Derlin-1 promotes the efficient degradation of the cystic fibrosis transmembrane conductance regulator (CFTR) and CFTR folding mutants. J. Biol. Chem..

[46] Biberstine-Kinkade K.J., DeLeo F.R., Epstein R.I., LeRoy B.A., Nauseef W.M., Dinauer M.C. (2001). Heme-ligating histidines in flavocytochrome b(558): identification of specific histidines in gp91(phox). J. Biol. Chem..

[47] Helmcke I., Heumüller S., Tikkanen R., Schröder K., Brandes R.P. (2009). Identification of structural elements in Nox1 and Nox4 controlling localization and activity. Antioxidants Redox Signal..

[48] Wu R.F., Ma Z., Liu Z., Terada L.S. (2010). Nox4-derived H_2_O_2_ mediates endoplasmic reticulum signaling through local Ras activation. Mol. Cell Biol..

[49] Denning G.M., Anderson M.P., Amara J.F., Marshall J., Smith A.E., Welsh M.J. (1992). Processing of mutant cystic fibrosis transmembrane conductance regulator is temperature-sensitive. Nature.

[50] Ren H.Y., Grove D.E., De La Rosa O., Houck S.A., Sopha P., Van Goor F., Hoffman B.J., Cyr D.M. (2013). VX-809 corrects folding defects in cystic fibrosis transmembrane conductance regulator protein through action on membrane-spanning domain 1. Mol. Biol. Cell.

[51] Chouchani E.T., James A.M., Fearnley I.M., Lilley K.S., Murphy M.P. (2011). Proteomic approaches to the characterization of protein thiol modification. Curr. Opin. Chem. Biol..

[52] Paulsen C.E., Carroll K.S. (2013). Cysteine-mediated redox signaling: chemistry, biology, and tools for discovery. Chem. Rev..

